# Service-Learning and Prosocial Competencies of Undergraduate Pre-service Teachers Toward Graduate Employability Skills

**DOI:** 10.12688/f1000research.163166.1

**Published:** 2025-06-13

**Authors:** Tolulope Victoria GBADAMOSI, Babatunde Kasim OLADELE

**Affiliations:** 1 Faculty of Education, Arts and Social Sciences Education Department, University of Ibadan, Ibadan, Oyo, Nigeria; 2 Faculty of Education, University of Johannesburg, Auckland Park 2006, Johannesburg, Gauteng, South Africa

**Keywords:** Presocial, Service-learning, Pre-service teachers, Student achievement

## Abstract

**Background:**

Many graduates are unprepared for the workforce because of the abysmal increase in unemployment rates. While academics have tried to incorporate employable skills into curricula, graduates must understand social and humanitarian principles to meet globalization demands. This study investigated how service-learning influences the prosocial skills of economics undergraduate pre-service teachers and its impact on their future employment prospects.

**Method:**

We adopted a mixed-method design of the sequential explanatory approach. A purposive random sampling technique was used to select 150 pre-service teachers (300 and 400 level) from five departments in the Faculty of Education at the University of Ibadan offering economics. A convenience sampling technique was used to sample 10 students for focus group discussions, who served as team leaders in service-learning courses. The Students’ Prosocial and Professional Competencies Questionnaire (r= 0.79) and a Focus Group Discussion Guide were used for data collection. Quantitative data were analyzed using mean, standard deviation and paired sample t-test at significance level of 0.05, while qualitative data were analyzed thematically.

**Results:**

The mean scores for the constructs were communication skills (2.61), teamwork (2.45), empathy (2.35), culture (2.30) and social responsibility (2.50). The mean for communication skills increased significantly from 2.61 before the intervention to 3.35. The reduction in the standard deviation indicates that the communication skills of pre-service teachers have become more consistent and prominent (F=24.87), confirming the statistical significance of these improvements. Similar improvements were observed in teamwork skills, with the mean score rising from 2.62 before intervention to 3.36 and statistically significant (F=9.71).

**Conclusion:**

The undergraduate students had low overall prosocial ability scores, indicating a lack of prosocial competencies essential for effective teaching and professional success in education. Policymakers and stakeholders should recognize the value of service learning and support its integration into teacher education programs through funding, resources, and institutional encouragement.

## Introduction

In Nigeria, there is significant concern regarding the employability of graduates, particularly in the education sector. Many graduates lack the essential soft skills required to thrive in a professional environment. The employment outcomes of graduates are crucial for university quality and curriculum relevance. However, high unemployment rates often leave graduates unprepared for the workforce, and employers perceive them as lacking core employability skills. Academics struggle to integrate employability skills into curricula, and globalization demands graduates with social and humane values. According to Ishokare and Gbadamosi (2020) and Odusanya and Omokhabi (2019), employers often cite deficiencies in communication, teamwork, and cultural competence among graduates. This gap necessitates educational interventions that can effectively bridge the gap between academic knowledge and practical skills.

Moreover, Nigeria’s educational policies increasingly emphasize inclusive education, which requires teachers to be adept at addressing the needs of students from various backgrounds, including those with special needs and marginalized communities. As the education sector continues to evolve, the demand for teachers who possess not only subject matter expertise, but also strong prosocial competencies has become increasingly apparent. Prosocial competencies, including empathy, cooperation, communication, and conflict resolution, are essential for creating a positive learning environment and fostering student engagement and development.

However, many pre-service teacher programs lack sufficient focus on developing the skills necessary for inclusive education. Mtawa, Fongwa, and Wilson-Strydom (2019); Olagoke-Oladokun; Mokhta; Gbadamosi; Dugguh (2020) noted that teachers often enter the workforce unprepared to create inclusive classrooms. The curriculum in some teacher education programs often does not align well with current educational demands and societal needs, resulting in teachers who may be knowledgeable in their subject areas but lack the interpersonal skills necessary to foster a supportive and inclusive classroom environment. Many pre-service teachers do not receive sufficient hands-on practice in actual classroom settings, limiting their ability to apply prosocial skills effectively ( Rocheleau, 2004; García Blanco, Sánchez, & Escudero, 2007; Wolff, Jarodzka, & Boshuizen, 2021).

Furthermore, a lack of robust support systems during training, including mentorship and counselling, can hinder the development of prosocial skills ( Mtawa, Fngwa, & Wilson-Strydom (2019). The competitive job market for teachers in Nigeria, coupled with economic constraints and policy issues within the education sector, further exacerbates these challenges ( Ishokare & Gbadamosi 2020). Collaboration with colleagues, parents, and the community is vital in modern educational settings. Teachers who lack prosocial competencies may struggle to work effectively in teams, collaborate on projects, or communicate with their parents and administrators. This can hinder their ability to contribute to school initiatives and negatively impact their reputation and career prospects ( Mtawa, Fngwa, & Wilson-Strydom, 2019). The absence of prosocial skills can increase teachers’ susceptibility to stress and burnout. Teachers who cannot effectively manage their emotions, resolve conflicts, or seek social support are more likely to experience professional dissatisfaction and leave their profession early. High turnover rates can be a red flag for potential employers, making it harder for these teachers to secure stable positions ( Wolff, Jarodzka, & Boshuizen, 2021). Teachers with strong prosocial competencies are more adaptable to change and continuous professional development. Lack of these skills can limit a teacher’s ability to respond to new educational demands, technological advancements, and innovative teaching methods, making them less competitive in the job market.

Given these challenges, ( Snell, Chan, Ma, & Chan, 2015) advocate experiential learning. Experiential learning is pivotal for bridging the gap between theoretical knowledge and practical classroom applications. These experiences significantly boost the employability of pre-service teachers by providing them with hands-on experience and practical skills necessary for teaching. Service-learning has emerged as a promising approach to experiential learning. Service-learning is recommended by in some studies to address a critical gap in teacher preparation programmes and supports the broader goal of inclusive education in Nigeria ( Olagoke-Oladokun; Mokhta; Gbadamosi; Dugguh, 2020). On the other hand, the Nigerian government has set various educational goals, including improving the quality of teaching and learning and ensuring equity and inclusion in education ( Mtawa, Fongwa, & Wilson-Strydom, 2019; Orlunga & Alikor, 2023) reported that the quality of teachers is the major determinant of the achievement of these laudable educational goals. He therefore suggested that, since Service-learning aligns with these goals by providing a framework through which pre-service teachers can engage with and contribute to their communities while developing essential professional skills, it should be adopted as an effective strategy for teacher education programs, thereby helping fulfill national priorities.

Service-learning integrates community services with academic instruction, emphasizing critical and reflective thinking, and personal and civic responsibility. This approach not only provides pre-service teachers with valuable practical experience but also fosters the development of essential prosocial skills ( Ajitoni & Gbadamosi, 2015; Olagoke-Oladokun; Mokhta; Gbadamosi; Dugguh 2020). Service-learning offers a practical solution by embedding real-world experiences into the academic curriculum, thereby enhancing pre-service teachers’ employability ( Mtawa, Fongwa, & Wilson-Strydom, 2019).

### Service-learning in teacher education

Service-learning bridges theoretical knowledge and practical experience, providing pre-service teachers with opportunities to apply their learning to real-world contexts. This pedagogical approach has been shown to enhance various soft skills crucial for employability. In the context of teacher education, service learning helps future teachers develop a deeper understanding of the communities they serve, fostering skills that are highly valued by employers. Baecher and Chung (2019) highlight that service-learning projects allow pre-service teachers to engage directly with diverse community groups, thereby increasing their cultural awareness and sensitivity. Cultural competence is essential in a multicultural society such as Nigeria, where teachers often work with students from various cultural backgrounds. The ability to navigate and respect these differences is a significant factor affecting employability.

### Prosocial competencies, service-learning and employability

Prosocial competencies, including empathy, teamwork, and effective communication are essential for creating supportive and inclusive learning environments. These competencies not only enhance classroom dynamics but also improve overall educational outcomes, making them highly sought after by education sector employers. Snell, Chan, Ma, and Chan (2015) found that service-learning experiences significantly improved intercultural communication skills among pre-service teachers. This improvement is particularly relevant in Oyo State, where schools are characterized by diverse student populations. Teachers who can effectively communicate across cultural boundaries are better equipped to meet the needs of all students, thereby enhancing their employability. Adigun and Ndwandwe (2023) emphasized the role of reflective practices in service learning in developing empathy. Reflective practices enable pre-service teachers to critically analyze their experiences and understand their own biases, leading to more empathetic and inclusive teaching practices. Empathy, as a prosocial competency, is crucial for building strong teacher-student relationships and fostering a positive classroom environment.

Mtawa, Fongwa, and Wilson-Strydom (2019) noted that service-learning projects that involve working with marginalized communities help pre-service teachers develop a sense of social responsibility and a commitment to inclusive education. These experiences are particularly valuable in Nigeria, where there is growing emphasis on inclusive practices in education. Employers in the Nigerian education sector increasingly seek graduates who demonstrate a commitment to equity and inclusion, making prosocial competencies developed through service learning a significant employability factor.

Mtawa, Fongwa, and Wilson-Strydom (2019) analyzed the role of service-learning in developing teamwork skills among pre-service teachers. The study found that collaborative projects in community settings require effective teamwork and fostering skills such as communication, coordination, and conflict resolution. These skills are crucial for employability as teaching often involves working in teams with other educators and staff. In another study, Baecher and Chung (2019) emphasized that service-learning projects offer pre-service teachers the opportunity to work closely with peers and community members. Immersive experience enhances interpersonal skills and collaborative learning, which are highly valued in educational settings. This study highlighted that employers seek teachers who can work well in team-oriented environments.

Olagoke-Oladokun, Hassan, and Atan (2018) investigated the development of empathy and team cohesion through service-learning. Their research indicated that working in diverse community settings fostered empathy, which is essential for effective teamwork. Team cohesion, developed through shared goals and experiences in service-learning projects, is another key employability skill that is highlighted in this study. Despite the recognized importance of prosocial competencies, traditional teacher education programmes often emphasize theoretical knowledge of practical skills. This gap between theory and practice can leave pre-service teachers underprepared for the classroom challenges. Integrating service-learning into teacher education can bridge this gap by providing hands-on experience that fosters essential skills. This study aims to explore the impact of service-learning on the prosocial competencies of economics undergraduate pre-service teachers in Oyo State, Nigeria, with a focus on enhancing their employability.

### Statement of the Problem

The traditional teacher education curriculum in Nigeria has been criticized for its lack of emphasis on practical skills and real-world applications. Consequently, many graduates enter the workforce without the prosocial competencies required for effective teaching. This study seeks to address this issue by examining the impact of service-learning on the development of prosocial competencies (communication skills, teamwork, empathy, cultural competency, and social responsibility) among economics undergraduate pre-service teachers. The goal is to determine whether integrating service learning into the curriculum can enhance these competencies and improve graduate employability. The objectives of this study were to assess the effectiveness of service-learning programmes in enhancing empathy, teamwork, and cultural competence in pre-service teachers and to analyze the relationship between developed prosocial competencies and pre-service teachers’ employability.

### Research Questions

The following research questions were addressed:

1. What is the level of prosocial competencies (empathy, teamwork, cultural competence) of economics undergraduate pre-service teachers before the intervention?
2. How do service-learning programmes impact the development of these prosocial competencies?

## Methods

The mixed-methods design of the sequential explanatory approach was employed to assess the effectiveness of service-learning on the prosocial skills of economics undergraduate pre-service teachers in Oyo State, Nigeria, and their relation to their future job prospects. The quantitative component involved 150 pre-service teachers at levels 300 and 400 from five departments within the Faculty of Education at the University of Ibadan, offering economics as their major or minor. For qualitative data, a sample of 10 students who served as team leaders in service-learning courses was selected for focus group discussion (FGD).

A validated questionnaire was used to assess prosocial abilities, which included the subconstructs of communication, teamwork, empathy, cultural competency, and social responsibility skills (r = 0.79), with a reliability coefficient established using Cronbach’s alpha. The study adhered to ethical considerations, providing comprehensive information about its objectives, methodologies, and potential advantages and disadvantages. The study included undergraduate students who provided written informed consent before data collection. The objectives, procedures, risks, benefits, and participants’ rights were explained. Enrollment in the study was optional, and the participants had the right to discontinue their involvement at any point without facing any repercussions. Ethical approval was obtained from the University of Ibadan Humanities and Social Sciences Ethics Committee (HSSEC) before initiating data collection.

The study was conducted over six months, from March 20, 2024, to September 27 **,** 2024. The instruments were administered at a convenient time for the participants. The FGD was conducted in two sessions, each lasting 45 minutes, approximately 90 minutes. Quantitative data collected were analyzed using mean, standard deviation and paired sample t-test at a significant level of 0.05, while the qualitative data were analyzed using thematic analysis, identifying major themes and patterns related to the influence of service-learning on participants’ prosocial competencies and employability.

## Results and Findings

Table 1 shows the level of prosocial competencies of economic undergraduates. The results show that the calculated weighted mean is 2.40, which is below the threshold of 2.5. This result implies that the undergraduates’ prosocial competencies were low. Specifically, based on the thresholds provided, all given skills fall under the low category. This suggests that the mean scores for communication skills, teamwork, empathy, cultural competence, and social responsibility were relatively low, indicating a need for improvement in this area.

**Table 1.** Level of Prosocial Competencies (Empathy, Teamwork, Cultural Competence) of Economics Undergraduate Pre-Service Teachers before the intervention.

S/N	Variables	N	Mean	StD	Remarks
1.	Communication Skills	150	2.61	0.59	Low
2	Teamwork	150	2.45	0.52	Low
3	Empathy	150	2.35	0.56	Low
4	Cultural Competence	150	2.30	0.59	Low
5	Social Responsibility	150	2.50	0.66	Low
Weighted mean = 2.44; Threshold = 2.50					

As shown in Table 2, the mean communication skills score increased significantly from 2.61 before the intervention to 3.35 after the intervention. The reduction in the standard deviation indicates that the communication skills of pre-service teachers became more consistent. The F-statistics of 24.868 and the p-value of 0.00 confirmed the statistical significance of these improvements. Similar improvements were observed in teamwork skills, as shown in Table 2, with the mean score rising from 2.62 to 3.36 and a statistically significant p-value of 0.002. The F-statistics of 9.718 and a p-value of 0.002 indicate that the improvement in teamwork skills after the service-learning intervention was statistically significant. A p-value of less than 0.05 confirms that the observed changes are unlikely to be due to chance.

**Table 2.** Effect of Service-Learning Programmes on the Development of Prosocial Competencies.

S/N	Variables	N	Pre Intervention		Post Intervention		F-Statistics	P-value (Sig.)
			Mean	St.D	Mean	St.D		
1.	Communication Skills	150	2.61	0.59	3.35	0.37	24.87	0.00
2	Teamwork	150	2.45	0.52	3.36	0.39	9.72	0.00
3	Empathy	150	2.35	0.56	3.28	0.43	3.59	0.06
4	Cultural Competence	150	2.30	0.59	3.29	0.42	6.12	0.01
5	Social Responsibility	150	2.50	0.66	3.35	0.47	20.26	0.000

Table 2 shows that the mean empathy skills score improved from 3.27 to 3.58, with a p-value of 0.059. While not statistically significant, the results suggest a positive impact of service learning on empathy, but it is not significant. This result is consistent with that of Adigun and Ndwandwe (2023), who emphasized the role of experiential learning in developing emotional intelligence. Cultural competency skills showed significant improvement, as indicated in Table 2, with the mean score increasing from 2.54 to 3.29 and a p-value of 0.014. A p-value of less than 0.05 confirms that the observed changes are very unlikely to be due to chance. Similarly, Table 2 shows that the mean social responsibility skills score increased significantly from 2.68 to 3.35, with a highly significant p-value of 0.000. A p-value of less than 0.05 confirms that the observed changes are very unlikely to be due to chance.

From the qualitative data analyzed, service-learning enhanced graduate employability skills and undergraduates’ capabilities, such as empathy, communication, teamwork, ICT skills, and other soft skills. SL can enhance graduate outcomes beyond training graduates for employment, foster human capabilities, and enhance the overall quality of education for graduate employability. Specifically, the following themes were derived from FGD.

**Communication Skills** A participant said his “ *Engaging in collective discussions throughout the service-learning project significantly enhanced his communication abilities.*” He smiles and says, “ *I acquired the skill of expressing my ideas with clarity and actively engaging in attentive listening to understand different viewpoints.*”

Another participant said her public speaking was improved as a result of her service-learning experience *“Delivering our study findings to the community enabled me to conquer my phobia of public speaking, which I worried about it long ago. Speaking in front of an audience has boosted my confidence, which will be advantageous in my teaching profession’”.*

***C*ollaboration Skills**

Meanwhile, *“the collaborative service-learning Project engaged me with my peers and has demonstrated the significance of teamwork.*" (Participant B). In another vein, Participant C said, “ *Collaborating on the project necessitated leveraging each other’s abilities to achieve success, and this encounter has enhanced my aptitude for working well in a team”.*

Collaborative Problem Solving: “ *Throughout the service-learning project, we encountered numerous obstacles. However, by collaboratively resolving these issues, we were able to cultivate robust teamwork abilities." Now I comprehend the criticality of collaboration in a teaching environment.*

**Empathy Skills**

Community Engagement is the ability to actively participate in and connect with the community, demonstrating understanding and compassion towards others. “Engaging with community members throughout the service-learning project facilitated my cultivation of a more profound understanding and ability to share in the emotions and experiences of others.” (Participant F). *I Listening to the narratives of my junior colleagues and comprehending their hardships in getting relevant course materials heightened my empathy and comprehension.*

Supportive Roles: “ *Participating in the service-learning programme and volunteering to develop course material google drives enhanced my ability to understand and relate to individuals from various backgrounds.” This experience has heightened my awareness and responsiveness to the requirements of my prospective pupils (Paticipant E). Another partcipants “Service-learning experience made me understand that teaching is not about academics but also about caring about students’ needs and adapting teaching methods with the students’ needs. service-learning allows me to interact with students from various home, it helps me to understand that each student has their unit challenges and developing an empathetic approach to their needs”*

Furthermore, Participant D said “ *I have acquired with have Skills to work with people through service-learning. The skill will enable me to work with people easily. This is what employers of labourers looking for. I have an advantage over my colleagues because of my participation in service-learning. In fact everyone in my team participated, I can work with my co-workers too”.*

**Cultural Competency Skills**: “Engaging in a service-learning program with people from different backgrounds greatly improved my cultural competency skills.” I have developed a deep appreciation and profound regard for the diverse cultural practices and beliefs.

**Cross-Cultural Communication Skill:** “Participating in the service-learning project enhanced my ability to effectively communicate with individuals from diverse cultural backgrounds.” I have developed a heightened understanding of cultural differences and acquired skills to relate to people.

**Social Responsibility Skills**: Community Service Projects: “Engaging in community service projects through the service-learning program cultivated a profound feeling of social responsibility. I have come to recognize the significant influence I can have through my activities and the crucial value of contributing to the community.

In another vein, Participant F said, *“Engaging in advocacy and awareness campaigns as part of the service-learning project enhanced my sense of social responsibility.*” The statement emphasized the significance of tackling societal problems and promoting constructive transformation.

**Broaden Teaching skills:** Another participant, when asked about how service-learning generally prepares her for a teachin *g job said “serivce-learning has influenced my experience because it takes me outside the classroom environment, I can also help others and impart knowledge outside of the classroom not limiting to my students within the classroom. it has broadened my knowledge in teaching, makes me see other side of teaching and other perspective, like doing voluntary activities to people in the community, like imparting knowledge into people and help them to be knowledgeable.*

**Saleable Skills**

Moreover, most of the participants said that all aspects of service-learning are valuable. For instance, a participant J said “Ha *! Ha! All aspect of service-learning is valuable, everything, I mean everything.* She smiles. *I have acquired skills that make me fit t job market.* Everything is valuable in service learning.

**ICT Skills**

Another person says, normally, “ *I have basic knowledge of computers but through service-learning, I was able to learn advanced ICT skills such as the use of Excel, PowerPoints, and Photoshop. In fact, I learnt a lot in service-learning”*

Thematic analysis of the beneficial effects of service-learning on several prosocial skills provided pre-service teachers with valuable experiences that enabled them to cultivate the crucial skills necessary for their professional development and marketability.

## Discussion

The study revealed low overall prosocial ability scores among undergraduate students, indicating a lack of prosocial competencies essential for effective teaching and professional success in education. This lack of communication skills, teamwork skills, empathy, cultural competence, and social responsibility contributes to a lack of cohesive and productive educational environments ( Olagoke-Oladokun, Mokhtar, Gbadamosi, & Dugguh, 2020; Ishokare & Gbadamosi, 2020; ( Mtawa, Fongwa, & Wilson-Strydom, 2019). Empathy is essential for creating supportive learning environments, and low empathy scores indicate limited capacity to comprehend and empathize with others’ emotions, potentially leading to negative interactions. Limited cultural competence suggests a lack of preparation to manage cultural differences, and undergraduates may not be well prepared to navigate diverse settings. Social responsibility, which involves a commitment to contributing positively to society and advocating for social justice, is also low, suggesting that undergraduates may not fully understand their role in promoting societal well-being and advocating for peers and communities. These findings have significant implications for teacher training programs, emphasizing the need for focused interventions to enhance these skills.

The significant improvement in communication skills among undergraduate pre-service teachers due to the service-learning program highlights the effectiveness of this pedagogical approach. Quantitative data from this study support these findings, as evidenced by the increase in the mean communication skills score. These findings align with previous research that emphasizes the benefits of experiential learning methods in developing essential soft skills. According to Giles and Eyler (2019), service learning provides students with opportunities to apply classroom knowledge in real-world contexts, enhancing their communication and interpersonal skills. The results of this study are consistent with Furco’s findings, demonstrating that hands-on community-based learning experiences effectively develop pre-service teachers’ communication abilities. In another study, Eyler and Giles (2021) noted that teachers who could communicate effectively with students, parents, and colleagues were better equipped to foster a positive learning environment and manage classroom dynamics. The significant improvement in communication skills observed in this study suggests that pre-service teachers who participate in service-learning programs are more likely to meet the communication demands of their future roles, thereby enhancing employability. The findings demonstrated that structured community interaction is critical for the development of communication skills for employability, particularly in the teaching profession.

According to Eyler and Giles (2021), service learning provides students with opportunities to apply classroom knowledge in real-world contexts, thereby enhancing their teamwork and collaboration skills. The results of this study support Eyler and Giles’s findings, demonstrating that hands-on, community-based learning experiences effectively develop pre-service teachers’ teamwork abilities. In addition, the significant improvement in teamwork skills observed in this study suggests that pre-service teachers who participate in service-learning programs are more likely to meet the collaboration demands of their future roles, thereby enhancing their employability, which is supported by Mtawa, Fongwa, & Wilson-Strydom (2019), who noted that teachers who can work well with others are better equipped to create a supportive learning environment and manage classroom dynamics.

The increase in empathy skills suggests that service-learning can positively influence pre-service teachers’ ability to understand and share the feelings of others. This improvement, although not statistically significant, is educationally meaningful and highlights the potential of service-learning in fostering emotional intelligence. While the p-value of 0.059 indicates that the results are close to statistical significance, the results suggest that service-learning contributes to the development of soft skills, such as empathy. This finding was confirmed by the participants during the FGD, who reported a tremendous improvement in their skills. Employers in the education sector highly value these skills, as they are essential for creating positive learning environments and fostering student well-being. Graduates with enhanced empathy skills are likely to be more competitive in the job market, as they can demonstrate their ability to connect with students and colleagues at a deeper emotional level ( Baecher, Laura & Chung, Samantha, 2019; Ajitoni & Gbadamosi, 2015).

Significant improvement in both cultural competency skills among undergraduate pre-service teachers as a result of the service-learning program highlights the effectiveness of this pedagogical approach, which is in alignment with some previous research, such as Eyler and Giles (2021) and Ajitoni and Gbadamosi (2015), and this study’s qualitative data that service-learning provides students with opportunities to engage with diverse communities, enhancing their cultural awareness and competency. The significant improvement in cultural competency skills observed in this study suggests that pre-service teachers who participate in service-learning programmes are more likely to meet the cultural demands of their future roles, thereby enhancing their employability.

Service-learning has been shown to foster a sense of social responsibility by encouraging students to engage in community activities. The results align with those of Adigun and Ndwandwe (2023) and Snell, Chan, Ma, and Chan (2015), who note that service learning fosters a sense of civic duty and community engagement. The significant improvement in social responsibility skills observed in this study and feedback from the qualitative data support these findings and demonstrate that service-learning effectively promotes social responsibility among pre-service teachers, which is important in preparing pre-service teachers for the job market.

## Conclusion

These findings have important consequences for teacher training programs, highlighting the need for targeted interventions to improve employability skills. Integrating service-learning into teacher education programs can offer hands-on opportunities to improve communication, collaboration, understanding, cultural proficiency, and ethical obligations. Service-learning is crucial for increasing graduates’ employability. It provides pre-service teachers with useful abilities, encourages reflective practice, encourages community involvement, creates teamwork, and improves soft skills. Embedding service-learning into academic programs can bridge the gap between theoretical knowledge and practical applications, preparing graduates more effectively for professional challenges. This study provides empirical evidence supporting the effectiveness of service learning on graduate employability, suggesting that teacher education programs in Nigeria and beyond should consider incorporating service learning components to enhance graduate outcomes. These improvements not only prepare employees for the complexities of the classroom but also make them more competitive and attractive to employers in the education sector. Integrating service learning into teacher education programs is crucial for producing well-rounded, capable, and employable graduates.

### Recommendations

i. Service-learning is a valuable tool in teacher education, providing pre-service teachers with practical experience that fosters essential soft skills. Universities should establish partnerships with local communities to create meaningful service learning opportunities.
ii. Faculty members should receive training on how to integrate service learning into their courses and facilitate reflective practices among students.
iii. Ongoing assessment and evaluation of service-learning programs are crucial to ensuring that they meet educational objectives and provide valuable experiences for students.
iv. Policymakers should recognize the value of service learning and support its integration into teacher education programs through funding, resources, and institutional encouragement.

## Ethics and consent

The study received ethical clearance from the University of Ibadan Humanities and Social Sciences Ethics Committee (HSSEC) on the 4 ^th^ of March 2024 with approval number UI/SSHREC/2024/0052, confirming compliance with established ethical standards for research involving human participants. This approval attested to our compliance with accepted ethical norms for research involving human beings and was consistent with the Declaration of Helsinki’s tenets, which regulate such investigations. The study included undergraduate students who provided written informed consent before data collection. Participants were informed about the purpose of the research, its voluntary nature, and the confidentiality of their responses. Confidentiality was maintained throughout the study, with responses securely stored in password-protected digital files accessible only to the research team. Participants voluntarily consented to participate, fully aware of their rights and the measures taken to protect them. The study procedures reflect the research team’s commitment to ethical practices and study integrity.

## Data Availability

Zenodo: Dataset on Service-Learning and Prosocial Competencies of Undergraduate Pre-service Teachers Toward Graduate Employability Skills, https://doi.org/10.5281/zenodo.15495980 ( Gbadamosi and Oladele, 2025). This project contains the following underlying data:
1. Prosocial data. Sav. Data are available under the terms of the Creative Commons Attribution 4.0 International license (CC-BY 4.0). Zenodo: Dataset on Service-Learning and Prosocial Competencies of Undergraduate Pre-service Teachers Toward Graduate Employability Skills, https://doi.org/10.5281/zenodo.15495980 ( Gbadamosi and Oladele, 2025). This project contains the following underlying data:
1.
SOCIAL_COMPETENCE_RESEARCH_INSTRUMENTAPPENDIX I 2.
SOCIAL_COMPETENCE_FGD_RESEARCH_INSTRUMENTAPPENDIX II Data are available under the terms of the Creative Commons Attribution 4.0 International license (CC-BY 4.0).
