# Supplementary material for: Service-Learning and Prosocial Competencies of Undergraduate Pre-service Teachers Toward Graduate Employability Skills
Source: F1000Res. 2026 Apr 16;14:584. Originally published 2025 Jun 13. [Version 4] doi: 10.12688/f1000research.163166.4 (PMC12910194; doi:10.12688/f1000research.163166.4)
Supplement: Peer review history

# Reviewer report: Reviewer response for version 3

**DOI:** 10.5256/f1000research.191202.r454388

**Published:** 2026-02-16

**Authors:** Robi Hendra

**Affiliations:** 1 Universitas Jambi, Muaro Jambi, Indonesia

This work has great potential and relevance. Service-based learning to enhance the prosocial skills of prospective teachers in Nigeria is timely. The mixed-methods sequential explanation approach is also conceptually sound. The current version requires significant changes.

 Comments 1

 This study lacks instrument validation evidence, a major limitation. The authors claim overall reliability (α = 0.79) without reporting Cronbach's alpha for each subconstruct, content validity, EFA, or CFA. This is important since the multidimensional instrument assesses prosocial competence. Without rigorous construct validation, all quantitative data is suspect.

 Comment 2

 Inconsistencies remain in the statistical analysis. The authors cite a "t-test of means" but provide the F-statistic. The language and tables differ (e.g., the pre-teamwork score was 2.45 vs. 2.62 in the prior version). The authors don't give effect sizes (Cohen's d) or 95% confidence intervals, making it difficult to judge the changes' practical robustness.

 Comment 3

 The quantitative sampling method (150 students from five departments) is unclear. Convenience, purposive, or stratified sampling? This ambiguity raises questions about selection bias and findings generalizability. The writers must describe the sampling process and sample demographics.

 Comment 4

 Little is known about the service learning intervention. Readers don't know the six-month activities, meeting duration and frequency, or reflection component. It is impossible to determine if stated improvements are due to the program without a clear and complete explanation of the intervention.

 Comment 5

 Only 10 team leaders were chosen for the focus group discussion (FGD), which may increase positive response bias. This qualitative sample is unrepresentative and biased toward engaged students. Expanding the qualitative sample or triangulating with individual interviews is advised.

 Comment 6

 Brief and opaque qualitative data theme analysis is presented. It doesn't explain inductive/deductive coding, theme generation, inter-coder reliability, or Lincoln & Guba's trustworthiness measurements (credibility, dependability, confirmability). This greatly devalues ​​qualitative findings.

 Comment 7

 The integration of quantitative and qualitative data is weak. Qualitative data simply "supports" quantitative conclusions, but in a sequential explanatory design, it should explain why and how changes occur, especially in empathy, which is scarcely significant. The authors must present together to improve mixed-methods integration.

 Comment 8

 Typographical errors sentences, repeated concepts, and inconsistent litter the discussion section. The authors must modify and restructure the discussion to be more brief, critical, and openly address the study's shortcomings (particularly empathy's minor statistical significance) and actual policy consequences.

Is the work clearly and accurately presented and does it cite the current literature?

Partly

If applicable, is the statistical analysis and its interpretation appropriate?

Partly

Are all the source data underlying the results available to ensure full reproducibility?

Partly

Is the study design appropriate and is the work technically sound?

Partly

Are the conclusions drawn adequately supported by the results?

Partly

Are sufficient details of methods and analysis provided to allow replication by others?

Partly

Reviewer Expertise:

Educational Management, Leadership, teacher learning, Digital technologi, digital technologies

I confirm that I have read this submission and believe that I have an appropriate level of expertise to confirm that it is of an acceptable scientific standard, however I have significant reservations, as outlined above.

# Reviewer report: Reviewer response for version 3

**DOI:** 10.5256/f1000research.191202.r436097

**Published:** 2025-11-27

**Authors:** Daniel Oyeniran, Mopelola Oyeniran

**Affiliations:** 1 The university of Alabama, Alabama, USA; 2 The University of Alabama (Ringgold ID: 8063), Tuscaloosa, Alabama, USA

I am still seeing University of Ibadan under abstract methods

Is the work clearly and accurately presented and does it cite the current literature?

Yes

If applicable, is the statistical analysis and its interpretation appropriate?

Yes

Are all the source data underlying the results available to ensure full reproducibility?

Yes

Is the study design appropriate and is the work technically sound?

Yes

Are the conclusions drawn adequately supported by the results?

Yes

Are sufficient details of methods and analysis provided to allow replication by others?

Partly

Reviewer Expertise:

Psychometrics, Scale development, Economics, Machine learning

We confirm that we have read this submission and believe that we have an appropriate level of expertise to confirm that it is of an acceptable scientific standard.

# Reviewer report: Reviewer response for version 2

**DOI:** 10.5256/f1000research.189690.r424855

**Published:** 2025-10-20

**Authors:** Daniel Oyeniran, Mopelola Oyeniran

**Affiliations:** 1 The university of Alabama, Alabama, USA; 2 The University of Alabama (Ringgold ID: 8063), Tuscaloosa, Alabama, USA

I found this paper to be highly valuable for other researchers and even those in charge of planning and developing curriculum for teacher educators. However, there is need to pay attention to few method and analytical approaches in the paper to ensure clarity and applicability of its findings.

 In the abstract under the background, the word "unprepared" seems inappropriate. It is the degree that supposed to prepare them, not the workforce. It could read: "Many graduates are discouraged about the labor market...." The statement should be reviewed in first paragraph of the introduction. It seems that the student selected for the focus group discussion were selected because of their position during the course. It might be more purposive instead of convenient, except there are many of them, from which 10 were selected as they are available.

 The authors should check some typographical errors in the manuscript, such as paragraph sentence five in paragraph six of the introduction.

 I don't think it is necessary to have a title on a single paragraph: Service-learning in teacher education.

 Is the description of the sample not too narrow for easy identification? You do not need to mention the university.

 Was there a factor analysis that showed five factors? The reliability reported is the overall I suppose.

 If you use some software for both quantitative and qualitative analysis they could be mentioned under methods even if there are no citations for them.

 I have not seen any discussion about the intervention introduced in this study. It is necessary to mention it, its timeline/duration, and other necessary information for the reader's understanding.

 For table 1, what was used to decide the remark? If its the threshold, the mean for communication was greater than 2.50.

Is the work clearly and accurately presented and does it cite the current literature?

Yes

If applicable, is the statistical analysis and its interpretation appropriate?

Yes

Are all the source data underlying the results available to ensure full reproducibility?

Yes

Is the study design appropriate and is the work technically sound?

Yes

Are the conclusions drawn adequately supported by the results?

Yes

Are sufficient details of methods and analysis provided to allow replication by others?

Partly

Reviewer Expertise:

Psychometrics, Scale development, Economics, Machine learning

We confirm that we have read this submission and believe that we have an appropriate level of expertise to confirm that it is of an acceptable scientific standard, however we have significant reservations, as outlined above.

# Reviewer report: Reviewer response for version 1

**DOI:** 10.5256/f1000research.179470.r395991

**Published:** 2025-08-13

**Authors:** Owolabi Paul Adelana

**Affiliations:** 1 Open University, Milton Keynes, UK

**Abstract**

 This study is timely, particularly in addressing the gap between higher education and employability in its context, and in the context of globalization in general. I also commend the integration of service-learning as a pedagogical tool to enhance prosocial skills. Overall, the study contributes to teacher education discourse but requires refinement for clarity and coherence. For instance, the abstract needs to clearly articulate the theoretical framework guiding the study and a more concise presentation of key findings. The sampling strategy appears somewhat inconsistent (purposive random versus convenience). Overall, the abstract is good.

**Introduction**

 The authors have presented a good and well-structured introduction, though a more succinct four-paragraph presentation would have been better. Overall, the introduction proposes service-learning as a practical solution aligned with national goals (this is good as also stated in the abstract). However, the introduction houses some lengthy sentences which if reworked, could give clearer articulation to the introduction. Also, the citation format needs to be consistent – for instance, in the second line of the fourth paragraph, a citation is written as  ( Mtawa, Fngwa, & Wilson-Strydom (2019), whereas the bracket before 2019 should have been a comma. Please, check all to ensure that they are well-presented. In addition, some long sentences might be rewritten to give conciseness to the introduction. An example of such sentence is: *The curriculum in some teacher education programs often does not align well with current educational demands and societal needs, resulting in teachers who may be knowledgeable in their subject areas but lack the interpersonal skills necessary to foster a supportive and inclusive classroom environment.*

 Paragraphs 3 and 4 of the introductions have given some kind of problem statement to the study. The standalone “Statement of the Problem” could be integrated into the main Introduction to give further standardized view to the Introduction.

**Methods**

 The methods section is well-structured, presented and robust. This section demonstrates methodological rigour through a sequential explanatory mixed-methods design. The use of both quantitative and qualitative data strengthens the study’s validity and depth. Sampling strategies are clearly described, though, as I pointed out in the review of the abstract, the combination of purposive random and convenience sampling could be better justified. Instrument reliability is adequately reported (r = 0.79), and ethical procedures are commendably thorough. The six-month data collection period and detailed analysis techniques (paired t-test and thematic analysis) are appropriate for the research aims. The methods section is concise and clearly written.

**Results and Findings**

**Table 2**

**“**Table 1 shows the level of prosocial competencies of economic undergraduates. The results show that the calculated weighted mean is 2.40….” If you check the table, the weighted average is written as “2.44”. Please, correct.

**Table 2**

 “Similar improvements were observed in teamwork skills, as shown in Table 2, with the mean score rising from 2.62…” Please, check the table because I could see “2.45”. Also, for teamwork, in the table P-value is written as “0.00” but in the explanation, it is written as “0.002”.

 Please, carefully check all the figures in the table regarding each skill and what is reported in the interpretation section of this table. There seems to be inconsistencies in the reporting of the figures.

**Under the Qualitative section:**

 Participants are note code-named for the statements under the following. Please check and correct: Communication Skills, Collaborative Problem Solving, Supportive Roles, Cultural Competency Skills, Cross-Cultural Communication Skill, Social Responsibility Skills, Broaden Teaching skills……Please check the others as well.

**Under Empathy skill:**

 Please, correct this grammatical issue - I Listening to the narratives of my junior colleagues and ……

**Discussion**

 The discussion section effectively interprets the findings and connects them to existing literature, reinforcing the value of service-learning in enhancing prosocial competencies. It clearly articulates how improvements in communication, teamwork, empathy, cultural competence, and social responsibility contribute to employability. However, the section is repetitive in some places (example shown below) and could benefit from better synthesis of ideas. I also suggest a more critical reflection on limitations and broader implications for policy and curriculum reform. I hope this will help to enhance this section’s scholarly depth presentation.

**Examples of Repetition**

**Communication Skills**

*“The significant improvement in communication skills among undergraduate pre-service teachers due to the service-learning program highlights the effectiveness of this pedagogical approach.”*

*“The significant improvement in communication skills observed in this study suggests that pre-service teachers who participate in service-learning programs are more likely to meet the communication demands of their future roles…”*

 I suggest that these two sentences repeating the same idea about communication skill should be merged. They could also be streamlined.

**Recommendation**

 I suggest that this section written in narrative prose format.

**Conclusion**

 Th conclusions the authors presented are well-supported by the results of this study. The conclusion needs to acknowledge the limited statistical significance in empathy gains and potential limitations in result generalization to strengthen the credibility and scholarship of the conclusion.

Is the work clearly and accurately presented and does it cite the current literature?

Yes

If applicable, is the statistical analysis and its interpretation appropriate?

Yes

Are all the source data underlying the results available to ensure full reproducibility?

Yes

Is the study design appropriate and is the work technically sound?

Yes

Are the conclusions drawn adequately supported by the results?

Yes

Are sufficient details of methods and analysis provided to allow replication by others?

Yes

Reviewer Expertise:

Education and Educational Technology, AIED, AI Ethics, STEAM/STEM Education.

I confirm that I have read this submission and believe that I have an appropriate level of expertise to confirm that it is of an acceptable scientific standard, however I have significant reservations, as outlined above.

# Response

**Published:** 2025-10-13

**Authors:** Babatunde Oladele

**Affiliations:** Institute of Education, University of Ibadan, Ibadan, Oyo, Nigeria

The figures presented in the explanation section and tables have been adjusted correctly. The missing name codes in the qualitative report section have been inserted, and the communication skills, collaborative problem-solving, supportive roles, cultural competency skills, cross-cultural communication skills, social responsibility skills, and broadened teaching skills have been addressed. All suggestions regarding the discussion, conclusion, and recommendation sections have been addressed.
